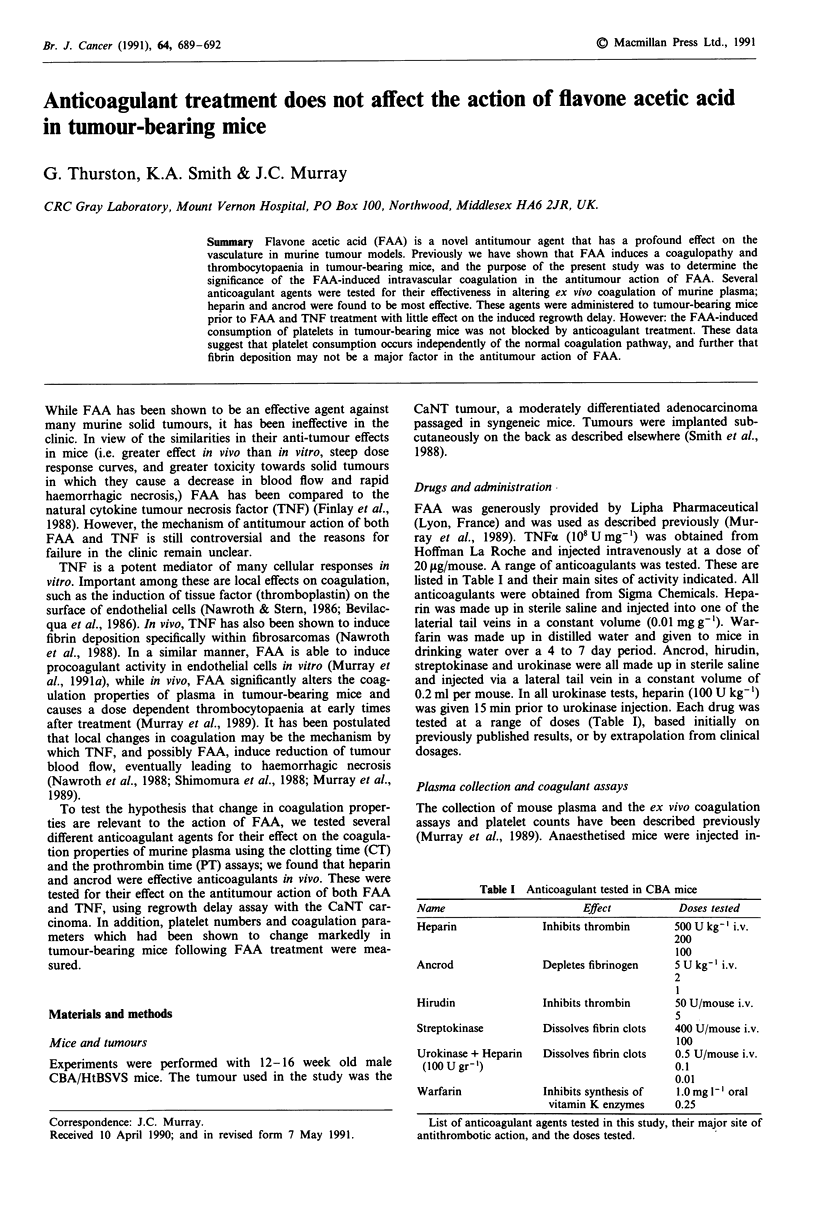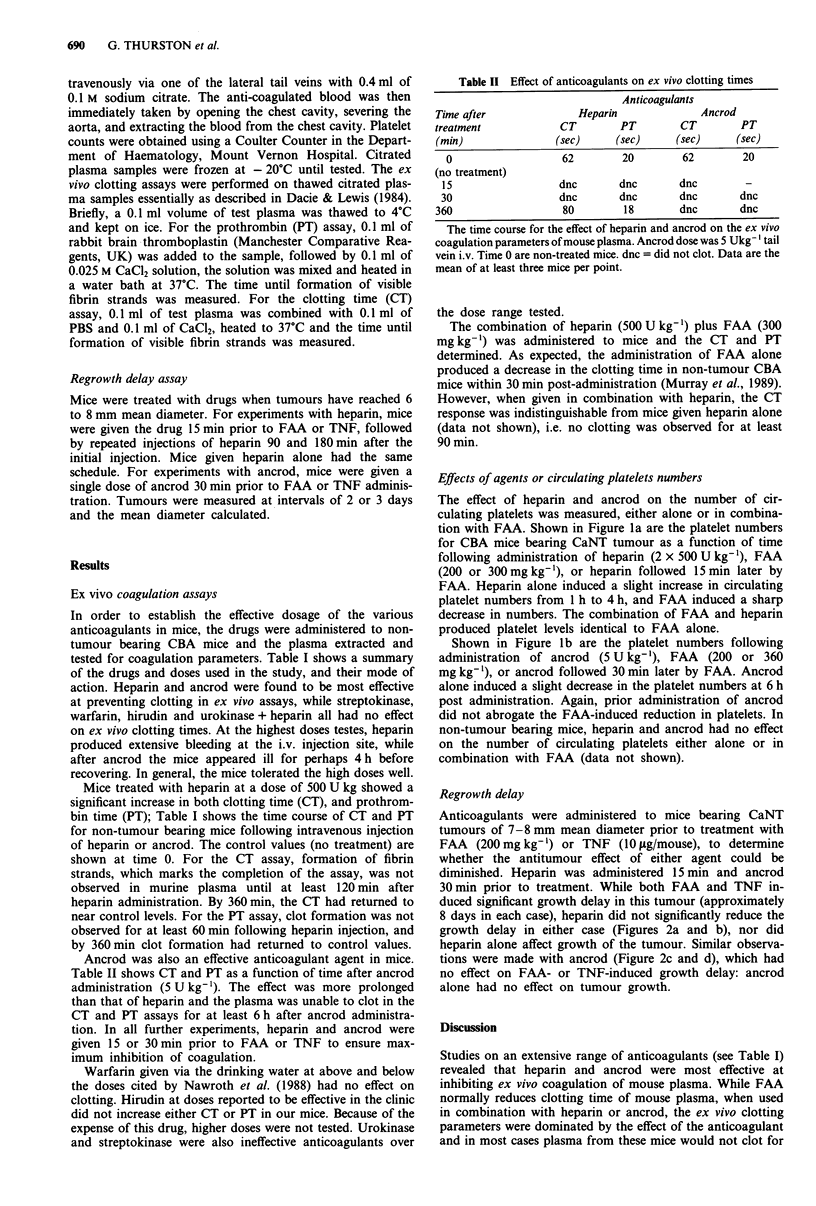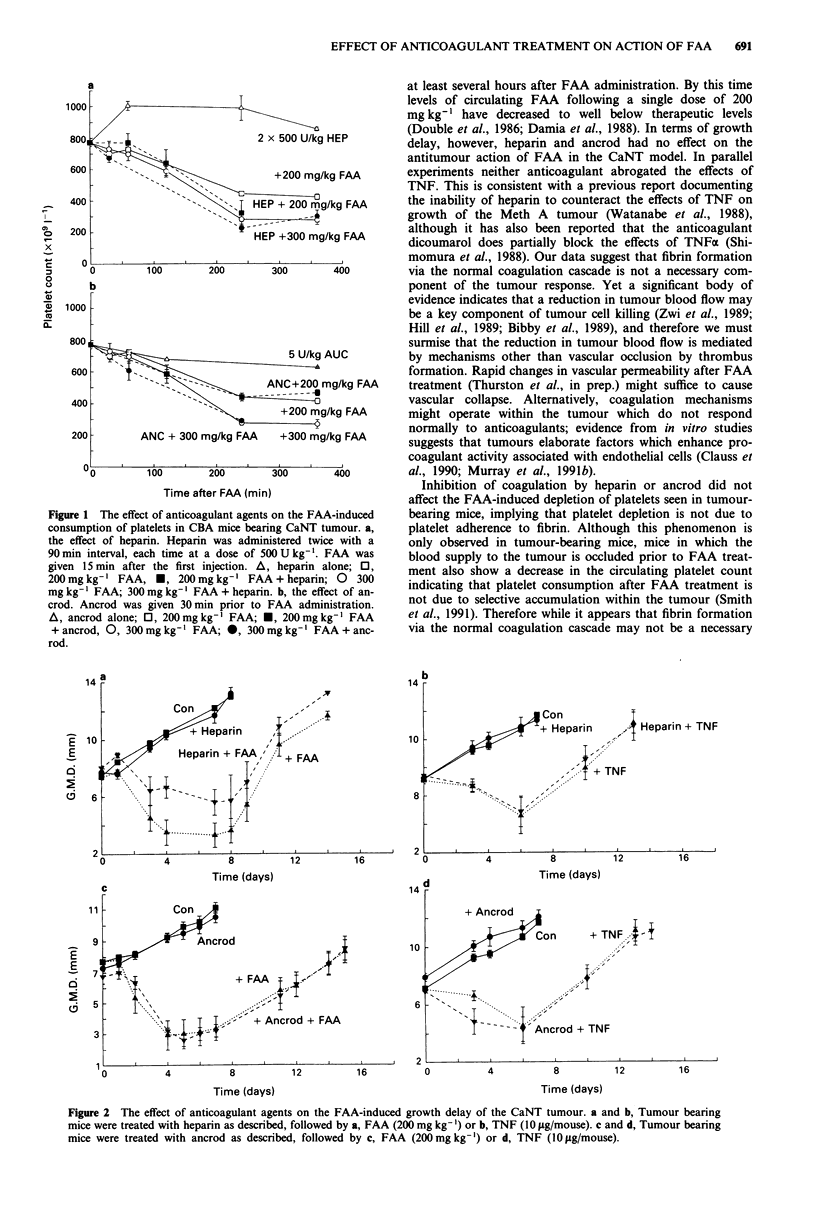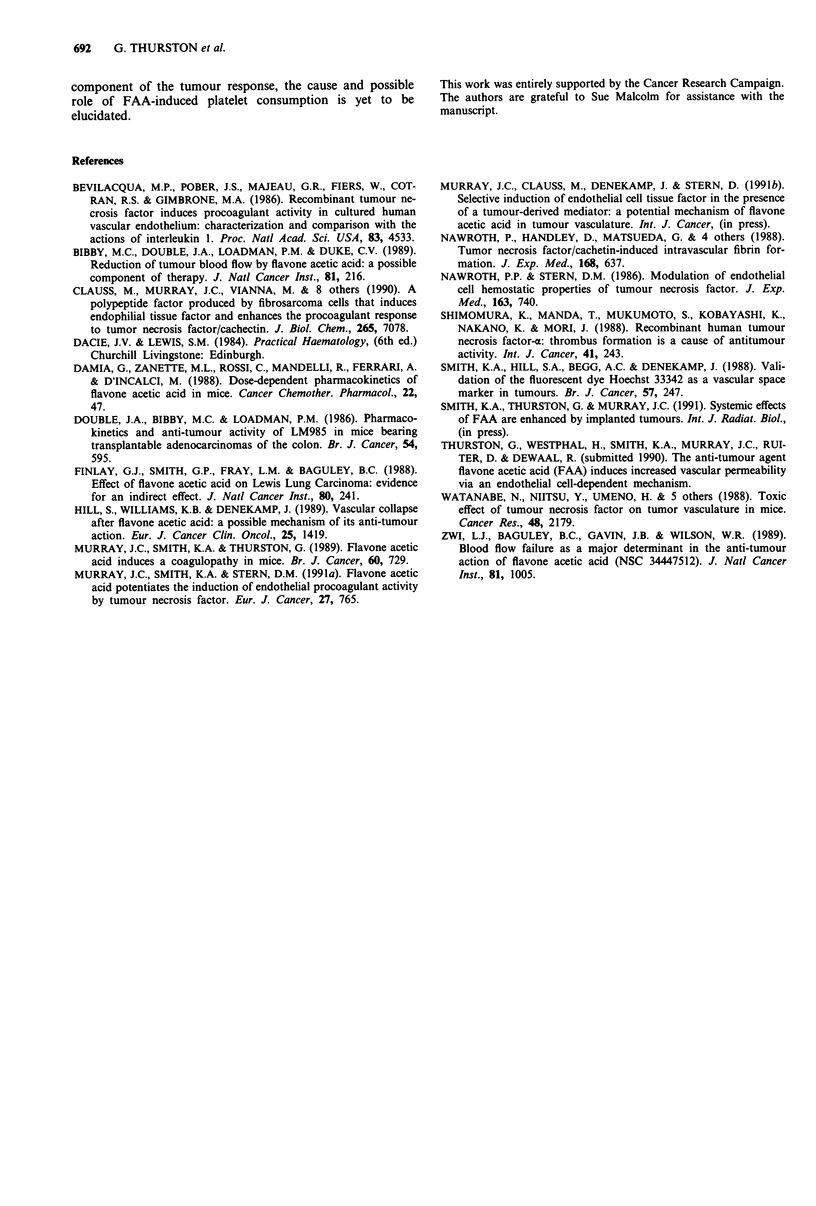# Anticoagulant treatment does not affect the action of flavone acetic acid in tumour-bearing mice.

**DOI:** 10.1038/bjc.1991.382

**Published:** 1991-10

**Authors:** G. Thurston, K. A. Smith, J. C. Murray

**Affiliations:** CRC Gray Laboratory, Mount Vernon Hospital, Northwood, Middlesex, UK.

## Abstract

Flavone acetic acid (FAA) is a novel antitumour agent that has a profound effect on the vasculature in murine tumour models. Previously we have shown that FAA induces a coagulopathy and thrombocytopaenia in tumour-bearing mice, and the purpose of the present study was to determine the significance of the FAA-induced intravascular coagulation in the antitumour action of FAA. Several anticoagulant agents were tested for their effectiveness in altering ex vivo coagulation of murine plasma; heparin and ancrod were found to be most effective. These agents were administered to tumour-bearing mice prior to FAA and TNF treatment with little effect on the induced regrowth delay. However: the FAA-induced consumption of platelets in tumour-bearing mice was not blocked by anticoagulant treatment. These data suggest that platelet consumption occurs independently of the normal coagulation pathway, and further that fibrin deposition may not be a major factor in the antitumour action of FAA.


Br. J. Cancer (1991), 64, 689-692 © Macmillan Press Ltd., 1991

### Anticoagulant treatment does not affect the action of flavone acetic acid in tumour-bearing mice

G. Thurston, K.A. Smith & J.C. Murray

*CRC Gray Laboratory, Mount Vernon Hospital, PO Box 100, Northwood, Middlesex HA6 2JR, UK.*

**Summary** Flavone acetic acid (FAA) is a novel antitumour agent that has a profound effect on the vasculature in murine tumour models. Previously we have shown that FAA induces a coagulopathy and thrombocytopaenia in tumour-bearing mice, and the purpose of the present study was to determine the significance of the FAA-induced intravascular coagulation in the antitumour action of FAA. Several anticoagulant agents were tested for their effectiveness in altering *ex vivo* coagulation of murine plasma; heparin and ancrod were found to be most effective. These agents were administered to tumour-bearing mice prior to FAA and TNF treatment with little effect on the induced regrowth delay. However: the FAA-induced consumption of platelets in tumour-bearing mice was not blocked by anticoagulant treatment. These data suggest that platelet consumption occurs independently of the normal coagulation pathway, and further that fibrin deposition may not be a major factor in the antitumour action of FAA.

While FAA has been shown to be an effective agent against many murine solid tumours, it has been ineffective in the clinic. In view of the similarities in their anti-tumour effects in mice (i.e. greater effect *in vivo* than *in vitro*, steep dose response curves, and greater toxicity towards solid tumours in which they cause a decrease in blood flow and rapid haemorrhagic necrosis,) FAA has been compared to the natural cytokine tumour necrosis factor (TNF) (Finlay *et al.*, 1988). However, the mechanism of antitumour action of both FAA and TNF is still controversial and the reasons for failure in the clinic remain unclear.

TNF is a potent mediator of many cellular responses *in vitro*. Important among these are local effects on coagulation, such as the induction of tissue factor (thromboplastin) on the surface of endothelial cells (Nawroth & Stern, 1986; Bevilacqua *et al.*, 1986). *In vivo*, TNF has also been shown to induce fibrin deposition specifically within fibrosarcomas (Nawroth *et al.*, 1988). In a similar manner, FAA is able to induce procoagulant activity in endothelial cells *in vitro* (Murray *et al.*, 1991*a*), while *in vivo*, FAA significantly alters the coagulation properties of plasma in tumour-bearing mice and causes a dose dependent thrombocytopaenia at early times after treatment (Murray *et al.*, 1989). It has been postulated that local changes in coagulation may be the mechanism by which TNF, and possibly FAA, induce reduction of tumour blood flow, eventually leading to haemorrhagic necrosis (Nawroth *et al.*, 1988; Shimomura *et al.*, 1988; Murray *et al.*, 1989).

To test the hypothesis that change in coagulation properties are relevant to the action of FAA, we tested several different anticoagulant agents for their effect on the coagulation properties of murine plasma using the clotting time (CT) and the prothrombin time (PT) assays; we found that heparin and ancrod were effective anticoagulants *in vivo*. These were tested for their effect on the antitumour action of both FAA and TNF, using regrowth delay assay with the CaNT carcinoma. In addition, platelet numbers and coagulation parameters which had been shown to change markedly in tumour-bearing mice following FAA treatment were measured.

### Materials and methods

### Mice and tumours

Experiments were performed with 12-16 week old male CBA/HtBSVS mice. The tumour used in the study was the CaNT tumour, a moderately differentiated adenocarcinoma passaged in syngeneic mice. Tumours were implanted subcutaneously on the back as described elsewhere (Smith *et al.*, 1988).

### Drugs and administration

FAA was generously provided by Lipha Pharmaceutical (Lyon, France) and was used as described previously (Murray *et al.*, 1989). TNFα ($10^8$ U mg$^{-1}$) was obtained from Hoffman La Roche and injected intravenously at a dose of 20 μg/mouse. A range of anticoagulants was tested. These are listed in Table I and their main sites of activity indicated. All anticoagulants were obtained from Sigma Chemicals. Heparin was made up in sterile saline and injected into one of the laterial tail veins in a constant volume (0.01 mg g$^{-1}$). Warfarin was made up in distilled water and given to mice in drinking water over a 4 to 7 day period. Ancrod, hirudin, streptokinase and urokinase were all made up in sterile saline and injected via a lateral tail vein in a constant volume of 0.2 ml per mouse. In all urokinase tests, heparin (100 U kg$^{-1}$) was given 15 min prior to urokinase injection. Each drug was tested at a range of doses (Table I), based initially on previously published results, or by extrapolation from clinical dosages.

### Plasma collection and coagulant assays

The collection of mouse plasma and the *ex vivo* coagulation assays and platelet counts have been described previously (Murray *et al.*, 1989). Anaesthetised mice were injected in-

**Table I** Anticoagulant tested in CBA mice

| *Name* | *Effect* | *Doses tested* |
|---|---|---|
| Heparin | Inhibits thrombin | 500 U kg$^{-1}$ i.v. |
| | | 200 |
| | | 100 |
| Ancrod | Depletes fibrinogen | 5 U kg$^{-1}$ i.v. |
| | | 2 |
| | | 1 |
| Hirudin | Inhibits thrombin | 50 U/mouse i.v. |
| | | 5 |
| Streptokinase | Dissolves fibrin clots | 400 U/mouse i.v. |
| | | 100 |
| Urokinase + Heparin (100 U gr$^{-1}$) | Dissolves fibrin clots | 0.5 U/mouse i.v. |
| | | 0.1 |
| | | 0.01 |
| Warfarin | Inhibits synthesis of vitamin K enzymes | 1.0 mg l$^{-1}$ oral |
| | | 0.25 |

List of anticoagulant agents tested in this study, their major site of antithrombotic action, and the doses tested.

Correspondence: J.C. Murray.
Received 10 April 1990; and in revised form 7 May 1991.

travenously via one of the lateral tail veins with 0.4 ml of 0.1 M sodium citrate. The anti-coagulated blood was then immediately taken by opening the chest cavity, severing the aorta, and extracting the blood from the chest cavity. Platelet counts were obtained using a Coulter Counter in the Department of Haematology, Mount Vernon Hospital. Citrated plasma samples were frozen at −20°C until tested. The *ex vivo* clotting assays were performed on thawed citrated plasma samples essentially as described in Dacie & Lewis (1984). Briefly, a 0.1 ml volume of test plasma was thawed to 4°C and kept on ice. For the prothrombin (PT) assay, 0.1 ml of rabbit brain thromboplastin (Manchester Comparative Reagents, UK) was added to the sample, followed by 0.1 ml of 0.025 M $CaCl_2$ solution, the solution was mixed and heated in a water bath at 37°C. The time until formation of visible fibrin strands was measured. For the clotting time (CT) assay, 0.1 ml of test plasma was combined with 0.1 ml of PBS and 0.1 ml of $CaCl_2$, heated to 37°C and the time until formation of visible fibrin strands was measured.

### *Regrowth delay assay*

Mice were treated with drugs when tumours have reached 6 to 8 mm mean diameter. For experiments with heparin, mice were given the drug 15 min prior to FAA or TNF, followed by repeated injections of heparin 90 and 180 min after the initial injection. Mice given heparin alone had the same schedule. For experiments with ancrod, mice were given a single dose of ancrod 30 min prior to FAA or TNF administration. Tumours were measured at intervals of 2 or 3 days and the mean diameter calculated.

### Results

### Ex vivo *coagulation assays*

In order to establish the effective dosage of the various anticoagulants in mice, the drugs were administered to non-tumour bearing CBA mice and the plasma extracted and tested for coagulation parameters. Table I shows a summary of the drugs and doses used in the study, and their mode of action. Heparin and ancrod were found to be most effective at preventing clotting in *ex vivo* assays, while streptokinase, warfarin, hirudin and urokinase + heparin all had no effect on *ex vivo* clotting times. At the highest doses testes, heparin produced extensive bleeding at the i.v. injection site, while after ancrod the mice appeared ill for perhaps 4 h before recovering. In general, the mice tolerated the high doses well.

Mice treated with heparin at a dose of 500 U kg showed a significant increase in both clotting time (CT), and prothrombin time (PT); Table I shows the time course of CT and PT for non-tumour bearing mice following intravenous injection of heparin or ancrod. The control values (no treatment) are shown at time 0. For the CT assay, formation of fibrin strands, which marks the completion of the assay, was not observed in murine plasma until at least 120 min after heparin administration. By 360 min, the CT had returned to near control levels. For the PT assay, clot formation was not observed for at least 60 min following heparin injection, and by 360 min clot formation had returned to control values.

Ancrod was also an effective anticoagulant agent in mice. Table II shows CT and PT as a function of time after ancrod administration (5 U $kg^{-1}$). The effect was more prolonged than that of heparin and the plasma was unable to clot in the CT and PT assays for at least 6 h after ancrod administration. In all further experiments, heparin and ancrod were given 15 or 30 min prior to FAA or TNF to ensure maximum inhibition of coagulation.

Warfarin given via the drinking water at above and below the doses cited by Nawroth *et al.* (1988) had no effect on clotting. Hirudin at doses reported to be effective in the clinic did not increase either CT or PT in our mice. Because of the expense of this drug, higher doses were not tested. Urokinase and streptokinase were also ineffective anticoagulants over the dose range tested.

**Table II** Effect of anticoagulants on *ex vivo* clotting times

| | *Anticoagulants* | | | |
|---|---|---|---|---|
| | *Heparin* | | *Ancrod* | |
| *Time after treatment (min)* | *CT (sec)* | *PT (sec)* | *CT (sec)* | *PT (sec)* |
| 0 (no treatment) | 62 | 20 | 62 | 20 |
| 15 | dnc | dnc | dnc | – |
| 30 | dnc | dnc | dnc | dnc |
| 360 | 80 | 18 | dnc | dnc |

The time course for the effect of heparin and ancrod on the *ex vivo* coagulation parameters of mouse plasma. Ancrod dose was 5 $Ukg^{-1}$ tail vein i.v. Time 0 are non-treated mice. dnc = did not clot. Data are the mean of at least three mice per point.

The combination of heparin (500 U $kg^{-1}$) plus FAA (300 mg $kg^{-1}$) was administered to mice and the CT and PT determined. As expected, the administration of FAA alone produced a decrease in the clotting time in non-tumour CBA mice within 30 min post-administration (Murray *et al.*, 1989). However, when given in combination with heparin, the CT response was indistinguishable from mice given heparin alone (data not shown), i.e. no clotting was observed for at least 90 min.

### *Effects of agents or circulating platelets numbers*

The effect of heparin and ancrod on the number of circulating platelets was measured, either alone or in combination with FAA. Shown in Figure 1a are the platelet numbers for CBA mice bearing CaNT tumour as a function of time following administration of heparin (2 × 500 U $kg^{-1}$), FAA (200 or 300 mg $kg^{-1}$), or heparin followed 15 min later by FAA. Heparin alone induced a slight increase in circulating platelet numbers from 1 h to 4 h, and FAA induced a sharp decrease in numbers. The combination of FAA and heparin produced platelet levels identical to FAA alone.

Shown in Figure 1b are the platelet numbers following administration of ancrod (5 U $kg^{-1}$), FAA (200 or 360 mg $kg^{-1}$), or ancrod followed 30 min later by FAA. Ancrod alone induced a slight decrease in the platelet numbers at 6 h post administration. Again, prior administration of ancrod did not abrogate the FAA-induced reduction in platelets. In non-tumour bearing mice, heparin and ancrod had no effect on the number of circulating platelets either alone or in combination with FAA (data not shown).

### *Regrowth delay*

Anticoagulants were administered to mice bearing CaNT tumours of 7–8 mm mean diameter prior to treatment with FAA (200 mg $kg^{-1}$) or TNF (10 μg/mouse), to determine whether the antitumour effect of either agent could be diminished. Heparin was administered 15 min and ancrod 30 min prior to treatment. While both FAA and TNF induced significant growth delay in this tumour (approximately 8 days in each case), heparin did not significantly reduce the growth delay in either case (Figures 2a and b), nor did heparin alone affect growth of the tumour. Similar observations were made with ancrod (Figure 2c and d), which had no effect on FAA- or TNF-induced growth delay: ancrod alone had no effect on tumour growth.

### Discussion

Studies on an extensive range of anticoagulants (see Table I) revealed that heparin and ancrod were most effective at inhibiting *ex vivo* coagulation of mouse plasma. While FAA normally reduces clotting time of mouse plasma, when used in combination with heparin or ancrod, the *ex vivo* clotting parameters were dominated by the effect of the anticoagulant and in most cases plasma from these mice would not clot for

**Figure 1** The effect of anticoagulant agents on the FAA-induced consumption of platelets in CBA mice bearing CaNT tumour. **a**, the effect of heparin. Heparin was administered twice with a 90 min interval, each time at a dose of 500 U kg$^{-1}$. FAA was given 15 min after the first injection. Δ, heparin alone; □, 200 mg kg$^{-1}$ FAA, ■, 200 mg kg$^{-1}$ FAA + heparin; O 300 mg kg$^{-1}$ FAA; 300 mg kg$^{-1}$ FAA + heparin. **b**, the effect of ancrod. Ancrod was given 30 min prior to FAA administration. Δ, ancrod alone; □, 200 mg kg$^{-1}$ FAA; ■, 200 mg kg$^{-1}$ FAA + ancrod, O, 300 mg kg$^{-1}$ FAA; ●, 300 mg kg$^{-1}$ FAA + ancrod.

at least several hours after FAA administration. By this time levels of circulating FAA following a single dose of 200 mg kg$^{-1}$ have decreased to well below therapeutic levels (Double *et al.*, 1986; Damia *et al.*, 1988). In terms of growth delay, however, heparin and ancrod had no effect on the antitumour action of FAA in the CaNT model. In parallel experiments neither anticoagulant abrogated the effects of TNF. This is consistent with a previous report documenting the inability of heparin to counteract the effects of TNF on growth of the Meth A tumour (Watanabe *et al.*, 1988), although it has also been reported that the anticoagulant dicoumarol does partially block the effects of TNFα (Shimomura *et al.*, 1988). Our data suggest that fibrin formation via the normal coagulation cascade is not a necessary component of the tumour response. Yet a significant body of evidence indicates that a reduction in tumour blood flow may be a key component of tumour cell killing (Zwi *et al.*, 1989; Hill *et al.*, 1989; Bibby *et al.*, 1989), and therefore we must surmise that the reduction in tumour blood flow is mediated by mechanisms other than vascular occlusion by thrombus formation. Rapid changes in vascular permeability after FAA treatment (Thurston *et al.*, in prep.) might suffice to cause vascular collapse. Alternatively, coagulation mechanisms might operate within the tumour which do not respond normally to anticoagulants; evidence from *in vitro* studies suggests that tumours elaborate factors which enhance procoagulant activity associated with endothelial cells (Clauss *et al.*, 1990; Murray *et al.*, 1991*b*).

Inhibition of coagulation by heparin or ancrod did not affect the FAA-induced depletion of platelets seen in tumour-bearing mice, implying that platelet depletion is not due to platelet adherence to fibrin. Although this phenomenon is only observed in tumour-bearing mice, mice in which the blood supply to the tumour is occluded prior to FAA treatment also show a decrease in the circulating platelet count indicating that platelet consumption after FAA treatment is not due to selective accumulation within the tumour (Smith *et al.*, 1991). Therefore while it appears that fibrin formation via the normal coagulation cascade may not be a necessary

**Figure 2** The effect of anticoagulant agents on the FAA-induced growth delay of the CaNT tumour. **a** and **b**, Tumour bearing mice were treated with heparin as described, followed by **a**, FAA (200 mg kg$^{-1}$) or **b**, TNF (10 μg/mouse). **c** and **d**, Tumour bearing mice were treated with ancrod as described, followed by **c**, FAA (200 mg kg$^{-1}$) or **d**, TNF (10 μg/mouse).

component of the tumour response, the cause and possible role of FAA-induced platelet consumption is yet to be elucidated.

This work was entirely supported by the Cancer Research Campaign. The authors are grateful to Sue Malcolm for assistance with the manuscript.

### References

BEVILACQUA, M.P., POBER, J.S., MAJEAU, G.R., FIERS, W., COTRAN, R.S. & GIMBRONE, M.A. (1986). Recombinant tumour necrosis factor induces procoagulant activity in cultured human vascular endothelium: characterization and comparison with the actions of interleukin 1. *Proc. Natl Acad. Sci. USA*, **83**, 4533.

BIBBY, M.C., DOUBLE, J.A., LOADMAN, P.M. & DUKE, C.V. (1989). Reduction of tumour blood flow by flavone acetic acid: a possible component of therapy. *J. Natl Cancer Inst.*, **81**, 216.

CLAUSS, M., MURRAY, J.C., VIANNA, M. & 8 others (1990). A polypeptide factor produced by fibrosarcoma cells that induces endophilial tissue factor and enhances the procoagulant response to tumor necrosis factor/cachectin. *J. Biol. Chem.*, **265**, 7078.

DACIE, J.V. & LEWIS, S.M. (1984). *Practical Haematology*, (6th ed.) Churchill Livingstone: Edinburgh.

DAMIA, G., ZANETTE, M.L., ROSSI, C., MANDELLI, R., FERRARI, A. & D'INCALCI, M. (1988). Dose-dependent pharmacokinetics of flavone acetic acid in mice. *Cancer Chemother. Pharmacol.*, **22**, 47.

DOUBLE, J.A., BIBBY, M.C. & LOADMAN, P.M. (1986). Pharmacokinetics and anti-tumour activity of LM985 in mice bearing transplantable adenocarcinomas of the colon. *Br. J. Cancer*, **54**, 595.

FINLAY, G.J., SMITH, G.P., FRAY, L.M. & BAGULEY, B.C. (1988). Effect of flavone acetic acid on Lewis Lung Carcinoma: evidence for an indirect effect. *J. Natl Cancer Inst.*, **80**, 241.

HILL, S., WILLIAMS, K.B. & DENEKAMP, J. (1989). Vascular collapse after flavone acetic acid: a possible mechanism of its anti-tumour action. *Eur. J. Cancer Clin. Oncol.*, **25**, 1419.

MURRAY, J.C., SMITH, K.A. & THURSTON, G. (1989). Flavone acetic acid induces a coagulopathy in mice. *Br. J. Cancer*, **60**, 729.

MURRAY, J.C., SMITH, K.A. & STERN, D.M. (1991*a*). Flavone acetic acid potentiates the induction of endothelial procoagulant activity by tumour necrosis factor. *Eur. J. Cancer*, **27**, 765.

MURRAY, J.C., CLAUSS, M., DENEKAMP, J. & STERN, D. (1991*b*). Selective induction of endothelial cell tissue factor in the presence of a tumour-derived mediator: a potential mechanism of flavone acetic acid in tumour vasculature. *Int. J. Cancer*, (in press).

NAWROTH, P., HANDLEY, D., MATSUEDA, G. & 4 others (1988). Tumor necrosis factor/cachetin-induced intravascular fibrin formation. *J. Exp. Med.*, **168**, 637.

NAWROTH, P.P. & STERN, D.M. (1986). Modulation of endothelial cell hemostatic properties of tumour necrosis factor. *J. Exp. Med.*, **163**, 740.

SHIMOMURA, K., MANDA, T., MUKUMOTO, S., KOBAYASHI, K., NAKANO, K. & MORI, J. (1988). Recombinant human tumour necrosis factor-α: thrombus formation is a cause of antitumour activity. *Int. J. Cancer*, **41**, 243.

SMITH, K.A., HILL, S.A., BEGG, A.C. & DENEKAMP, J. (1988). Validation of the fluorescent dye Hoechst 33342 as a vascular space marker in tumours. *Br. J. Cancer*, **57**, 247.

SMITH, K.A., THURSTON, G. & MURRAY, J.C. (1991). Systemic effects of FAA are enhanced by implanted tumours. *Int. J. Radiat. Biol.*, (in press).

THURSTON, G., WESTPHAL, H., SMITH, K.A., MURRAY, J.C., RUITER, D. & DEWAAL, R. (submitted 1990). The anti-tumour agent flavone acetic acid (FAA) induces increased vascular permeability via an endothelial cell-dependent mechanism.

WATANABE, N., NIITSU, Y., UMENO, H. & 5 others (1988). Toxic effect of tumour necrosis factor on tumor vasculature in mice. *Cancer Res.*, **48**, 2179.

ZWI, L.J., BAGULEY, B.C., GAVIN, J.B. & WILSON, W.R. (1989). Blood flow failure as a major determinant in the anti-tumour action of flavone acetic acid (NSC 34447512). *J. Natl Cancer Inst.*, **81**, 1005.